# Using Machine Learning Methods to Predict Hospitalization Based on Brixia Score and Patient Clinical Data (from the COVID-19 Pandemic)

**DOI:** 10.3390/medicina62020392

**Published:** 2026-02-17

**Authors:** Mirela Juković, Aleksandra Mijatović, Radmila Perić, Ljiljana Dražetin, Dijana Nićiforović, Dejan B. Stojanović

**Affiliations:** 1 Medical Faculty, University of Novi Sad, Hajduk Veljkova 3, 21000 Novi Sad, Serbia; radmila.peric@mf.uns.ac.rs (R.P.); ljiljana.drazetin@uns.ac.rs (L.D.); dijana.niciforovic@mf.uns.ac.rs (D.N.); 2 Centre for Radiology, University Clinical Centre of Vojvodina, Hajduk Veljkova 2, 21000 Novi Sad, Serbia; aleksandra.mijatovic@kcv.rs; 3 Institute of Lowland Forestry and Environment, University of Novi Sad, Antona Cehova 13d, 21000 Novi Sad, Serbia; dejan.stojanovic@uns.ac.rs

**Keywords:** hypertension, diabetes, machine learning, chest X-ray, Decision Tree, Logistic Regression, Random Forest, Support Vector Machine

## Abstract

*Background and Objectives:* The use of a standard chest X-ray has become a routine diagnostic method in daily clinical practice for the evaluation of a wide range of lung diseases. During the COVID-19 pandemic, significant challenges occurred in achieving accurate diagnostics and selecting appropriate therapies for patients with different symptoms of diseases. The aim was to cross-correlate radiological findings and clinical data and to develop models to predict hospitalization status, while evaluating the prognostic importance of the different variables. *Materials and Methods:* A set of variables including Brixia score, and clinical data: gender, age, hypertension, and diabetes was used to explore their association with patient hospitalization. Four different machine learning (ML) methods (*Decision Tree*—DT, *Logistic Regression*—LR, *Random Forest*—RF and *Support Vector Machine*—SVM) were used for hospitalization outcome prediction. *Results:* SVM appeared to be with the highest AUC (0.851), with low sensitivity, while DT was the most balanced in the context of AUC, accuracy, sensitivity, and specificity. Brixia score appeared to be the most important predictor for hospitalization within the group of predictors (gender, age, hypertension and diabetes). *Conclusions:* All four ML models that used in this study provided “good” prediction capabilities (AUC > 0.8), with the exception of SVM that had low sensitivity, emphasizing Brixia score as the strongest predictor of hospitalization. Application of ML methods have considerable potential in various aspects of medical clinical practice and future studies could potentially indicate the importance of applying the ML model in more precise diagnosis, therapy and prognosis of the patient’s clinical condition.

## 1. Introduction

During the COVID-19 pandemic, identifying lung changes that would confirm the presence of inflammation was a daily challenge for radiologists. Pathological patterns on chest radiograms were often subtle; however, by integrating clinical data and comorbidities, clinicians were able to determine which patients required hospitalization and which could be safely managed in an outpatient setting. The semi-quantitative Brixia score, which was used to detect changes in the lung parenchyma, has the potential to serve as a valuable radiographic database for applications in artificial intelligence. Machine learning (ML), as a branch of AI, is now widely applied in many areas of diagnostic radiology, where it assists in learning patterns that predict outcomes and improve decisions regarding patient treatment [1,2,3,4]. The COVID-19 pandemic caused numerous pneumonia cases. The role of radiologists became increasingly important given the rising number of lung examinations. Thoracic imaging that includes chest X-ray, computed tomography (CT) and computed tomography angiography (CTA) has importance in diagnostics, monitoring and therapy in patients with COVID-19 pneumonia and potential complications. For the importance of radiological methods in the detection of COVID-19 cases, particularly, CT was recognized in early stages of pandemics [5]. However,, the wide availability and rapid execution give preference of chest X-ray over CT in daily clinical practice [6]. In the light of the COVID-19 crisis, *Borghesi and Maroldi* (*2020*) [7] designed the new scoring system for evaluation of the lung abnormalities on the chest X-ray called Brixia score. Such scoring may be useful in clinical practice, as well as for the application of ML methods. ML modeling through the prism of various state-of-the-art techniques in this study was applied to standard chest radiographs and clinical data of patients.

The aims of this article were as follows:

(i) To evaluate pairwise associations between admission chest X-ray Brixia radiographic score, age, gender, selected comorbidities (hypertension, diabetes, and all reported comorbidities), hospitalization status and length of hospital stay in patients with COVID-19 infection, using correlation coefficients.
(ii) To develop and compare the performance of four machine learning classification models (*Decision Tree*, *Logistic Regression*, *Random Forest*, and *Support Vector Machine*) using available predictors (Brixia score, age, gender, hypertension, and diabetes) to predict the binary outcome of hospitalization (yes/no).
(iii) To quantify and compare the relative importance of the Brixia score in relation to other predictors across the different models.

## 2. Materials and Methods

### 2.1. Patient’s Group and Study Design

All patients in the study were admitted to the University Clinical Centre of Vojvodina (UCCV)-regional clinical center that covers population of about 2 million, after triage and local health centers and hospitals as they were suspected of severe disease cases. The study is retrospective and included 222 patients who had treatment or were hospitalized in UCCV, during the second wave of COVID-19 epidemic in Serbia (July–August 2020) (Table S1). Data was collected using Radiology Information System (RIS), Picture Archiving and Communication System (PACS) and Clinical Information System (CIS). An initial chest X-ray examination was the first line of diagnostic method for evaluation of potentially chest COVID-19 infections. The chest X-ray on admission of all 222 patients included in the study was interpreted by one radiologist with twelve years of experience, using the Brixia score [8] from 0 to 18 value. Each lung was divided into three regions: upper, middle region and base. Parenchymal findings include normal (0 value), interstitial changes (1 value), combination of interstitial and alveolar findings with interstitial dominance (2 value) and combination of interstitial and alveolar changes with alveolar dominance (3 value). The values for each zone of the chest were added together to obtain the final score number.

Gender of patients, age, comorbidities (hypertension, diabetes, and others included altogether under all comorbidities variable), hospitalization and the number of hospital days were cross correlated with Brixia score and among each other. Brixia score is a scoring system created to describe clinical grading of chest X-ray in patients with COVID-19 infection. Classification model was created based on hospitalization status (yes/no). It was assumed as the type of outcome after initial X-ray diagnostics at admission. Patients who were not initially hospitalized, but later experienced clinical deterioration and subsequently hospitalized, were not included in the study. Death cases were relatively rare to be considered as statistically relevant outcome in the sense of observed patient group. Hospitalization days as the proxy of illness severity were considered for making regression model, but that was not justified since about fourth of hospitalized patients after health improvement were transferred to local hospitals, after which data about the remaining time that they spent in hospital were not available.

### 2.2. Statistical Analysis

Collected data were analyzed using *R statistical language* (version 4.5.2). Specifically, packages *ggplot2* (4.0.1), *corrplot* (0.95), *caret* (7.0.1), *randomForest* (4.7.1.2), *pROC* (1.19.0.1), *rpart* (4.1.24), and *e1071* (1.7.17) were used for exploring data, machine learning modeling and visualization. ML methods: *Decision Tree*—DT, *Logistic Regression*—LR, *Random Forest*—RF and *Support Vector Machine*—SVM were used in this study. All four techniques are used for classification.

DT splits data into branches based on feature thresholds until reaching a decision, LR estimates probabilities using the logistic (sigmoid) function, RF groups many decision trees, each trained on random subsets of data/features, while SVM finds the optimal hyperplane that maximizes the margin between classes. They are a good solution when you have numerical and categorical data such as the case with patient data. Dependent variable was hospitalization status (YES/NO). Independent variables were Brixia Score, gender, age, presence of comorbidities. The formula was HOSPITALIZED ~ BRIXIA.SCORE + GENDER + AGE + HYPERTENSION + DIABETES. Variable ALL.COMORBIDITIES were omitted from the formula since hypertension and diabetes were the most frequent comorbidities in the patient’s group. Five predictors were taken for practical reasons. Five predictors were available for all subjects in the dataset, but it also fulfils the criteria of at least 10 up to 25 events per predictor (EPV) [8]. In our case, we had 185 hospitalized patients, meaning that we were with five predictors in a safe zone (185/5 = 37 EPV). Due to the relatively small sample, more predictors would increase chances of overfitting, unstable coefficients and high variance.

We calculated Pearson’s correlation coefficients for all pair-wise relationships between variables. Included also were binary predictors (e.g., hypertension, diabetes, and all comorbidities) and the binary outcome (hospitalized: yes/no). Although Pearson’s r is usually applied to continuous data, it is mathematically equal to the point-biserial correlation when one variable is continuous and the other is dichotomous, and to the phi coefficient when both are dichotomous. In practice, Pearson’s r is frequently used in clinical and epidemiological studies for mixed-type data (continuous, binary, and ordinal) because it is straightforward and interpretable as a standardized measure of linear association (range –1 to +1).

### 2.3. Machine Learning Workflow

The overall dataset (*n* = 222) was split into training and testing sets using a 80:20 ratio (178 training samples/44 test samples). The split was performed randomly but stratified by the target variable (hospitalized: yes/no) to preserve the original class distribution (~83% hospitalized) in both sets, using function *createDataPartition* (*caret* package, ver. 7.0.1). All preprocessing steps were performed after splitting to avoid data leakage.

Binary predictors (hypertension, diabetes) were represented as 0/1. Gender was modeled as a categorical variable and was dummy or 0–1 encoded for algorithms requiring numeric input. For LR and SVM, continuous features (age, Brixia score) were standardized using z-score transformation fitted exclusively on the training set and applied to the test set. No scaling was performed for tree-based models (*RF*, *DT*). Hyperparameter tuning was conducted using 5-fold cross-validation. The best model configuration was chosen using highest cross-validated AUC.

Feature importance was extracted using methods specific for the model: coefficient absolute values for LR, permutation importance for RF, and Gini impurity decrease for DT. Since those metrics are on different scales and reflect different notions of importance (coefficient magnitude vs. predictive contribution vs. node purity), direct numerical comparison is not intended in the first place. What we did was model min–max normalization to a common 0–100 scale to allow comparison of relative ranking and consistency across model algorithms.

## 3. Results

### 3.1. Patients Overview

**Figure 1:**
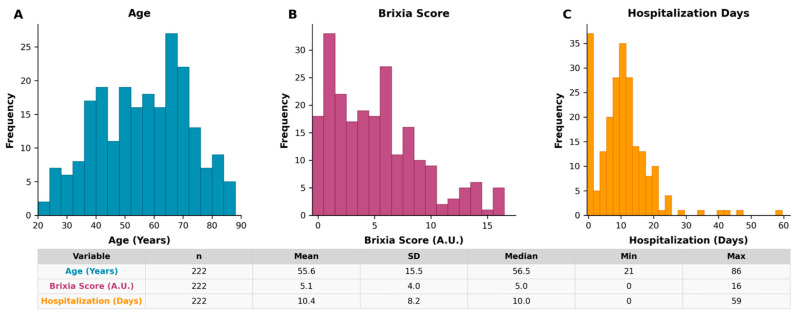
Frequency distributions of (**A**) patient age (years), (**B**) Brixia score (A.U. = arbitrary units ranging from 0 to 18), and (**C**) length of hospitalization (days) among COVID-19 patients (n = 222) admitted to the University Clinical Centre of Vojvodina during the second wave (July–August 2020). Summary statistics are provided in the table below.

**Figure 2:**
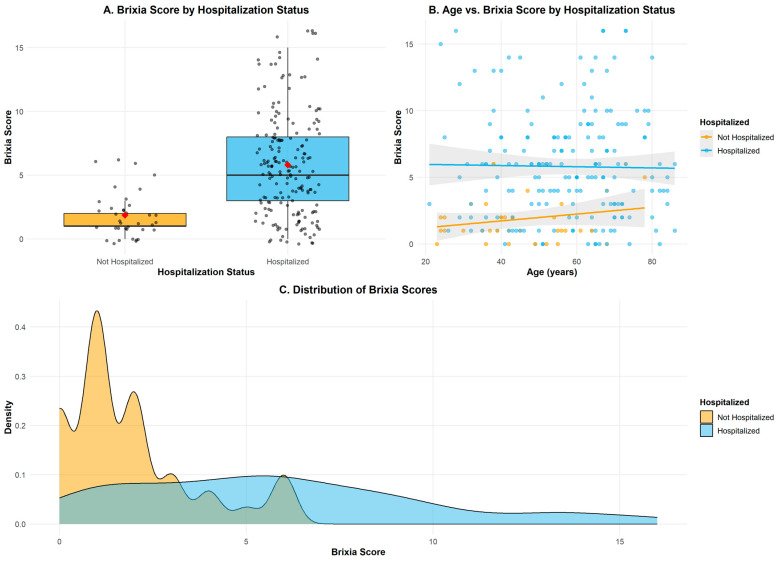
Brixia score distribution among hospitalized and non-hospitalized COVID-19 patients, with age–score relationship and density estimation (patient data from University Clinical Centre of Vojvodina, July–August 2020). (**A**) Boxplot of Brixia score by hospitalization status; individual grey dots represent single patient observations and the red diamond indicates the group mean. (**B**) Scatter plot of Brixia score versus age (years). Each dot represents an individual patient, colored by hospitalization status, orange—not hospitalized and blue—hospitalized. Solid lines represent linear regression fits for each group, and the grey shaded areas represent the 95% confidence intervals. (**C**) Kernel density plot of Brixia score distributions by hospitalization status, orange—not hospitalized and blue—hospitalized. The y-axis (“Density”) is expressed in probability density units.

The most common symptoms of COVID-19 among observed patients were fever, a cough, and muscle weakness. Among 222 patients, 155 were males and 67 females. Patients were between 21 and 86 years old. Mean age of the patient group was 55.59 ± 15.54 years. Males had 55.15 ± 15.14 years, while females had 56.58 ± 16.48 year in average. Some of the comorbidities (diabetes, hypertension, obesities, asthma, cardiomyopathy, cancer, leukaemia, melanoma, Alzheimer’s disease, Parkinson’s disease, Down’s syndrome, cystic fibrosis, and rheumatoid arthritis) occurred in 108 subjects. Hypertension and diabetes, as the most common comorbidities, were present in 76 and 31 patients, respectively. Average Brixia score acquired from the lung chest X-ray was ~5 (Figure 1 and Figure 2). Mortality was observed in 8 patients (seven males and one female) with the average age ~71.

### 3.2. Hospitalization Status

**Figure 3:**
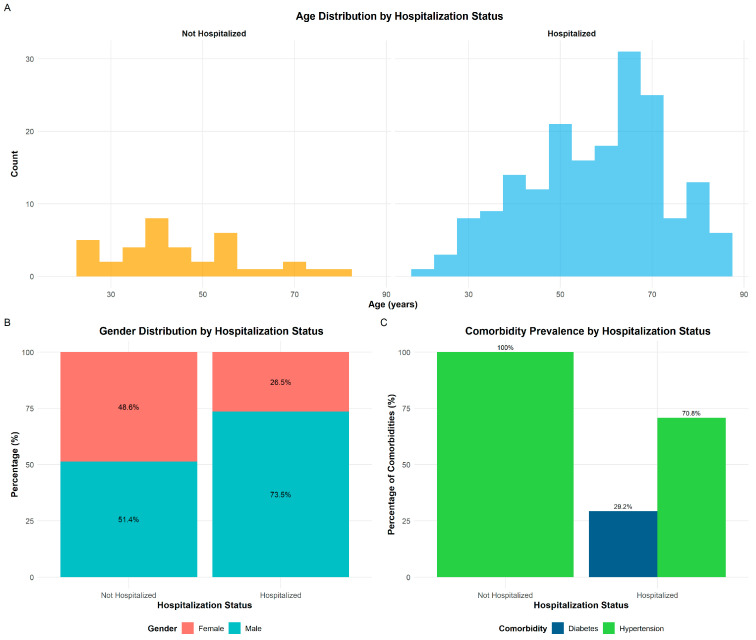
Patient characteristics of the study cohort by hospitalization status among COVID-19 patients (*n* = 222) at the University Clinical Centre of Vojvodina during the second wave (July–August 2020). (**A**) Age distribution (years) stratified by hospitalization status (left: not hospitalized, n = 37; right: hospitalized, n = 185), (**B**) Gender distribution (%) within each hospitalization group; (**C**) Prevalence of the two most common comorbidities—hypertension (green) and diabetes (dark blue)—by hospitalization status, expressed as the percentage of patients within each group.

There were 185 hospitalized patients (185/222), among which 45 initially were examined, started treatment from home, and some days later were hospitalized after health deterioration. Roughly 88% (136/155) of males and 73% (49/67) of treated female patients were hospitalized (Figure 3). Among the hospital-treated patient group, the share of males was ~74% (136/185), while among gender/hospitalization sub-groups, males were 15% more often hospitalized than females. Having in mind the overlapping average age and standard deviations, the age difference between sexes may not be statistically significant. Maximal hospitalization lasted 59 days, while average time spent in hospital was ~12 days.

The average age of hospitalized patients was ~58 years while the age of non-hospitalized were ~45 years. The average age of hospitalized male patients was ~56 years while the age of non-hospitalized males was ~48 years. The average age of hospitalized female patients was ~62 years while the age of non-hospitalized females was ~42 years.

There were 108 patients with some comorbidity. Only two people from the group with comorbidities were treated without admission to the hospital. Although comorbidities were crucial factors for clinicians to make decisions about hospitalization, considerable number of previous healthy people were hospitalized (79/114, with average age of ~52 years). The average Brixia score of hospitalized patients was ~6 and for non-hospitalized ~2. Average Brixia score of hospitalized male patients was ~6 and for non-hospitalized, ~2, while *score* for hospitalized female patients were ~5 and for non-hospitalized, ~2. Forty-four patients with health improvement after spending ~11 days in a clinical center were moved to local hospitals, which increases the average time that the hospitalized patient group spent in hospital for an unknown time. The eight patients who died also spent an average ~11 days in hospital.

### 3.3. Correlation

**Figure 4:**
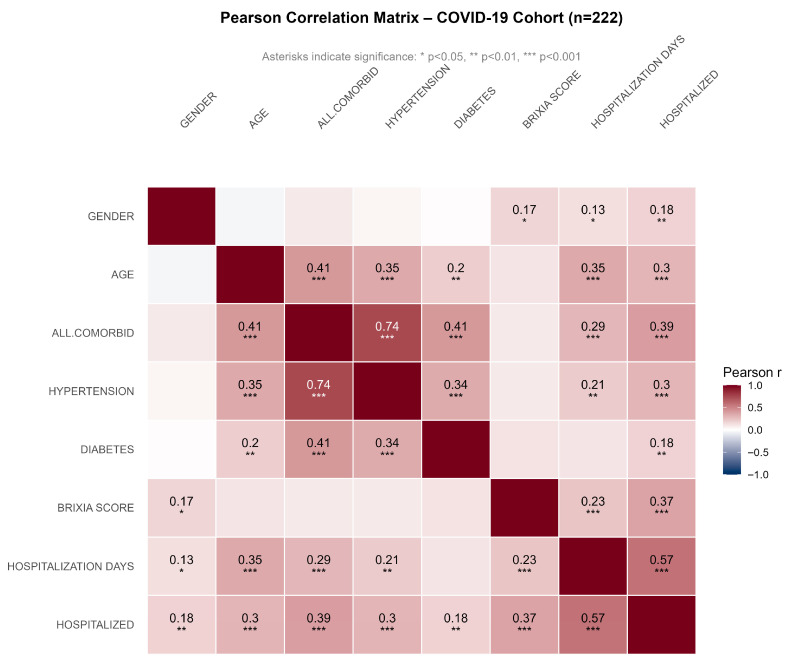
Heatmap of correlation among age, Brixia score, hospitalization days, gender, comorbidities and hospitalization among COVID-19 patients in University Clinical Centre of Vojvodina during the second wave (July–August 2020) (presented are Person correlation coefficients that are equivalent to point-biserial correlation).

Pearson’s correlation coefficients are displayed only for associations between variables that were statistically significant (*p* < 0.05) (Figure 4). Non-significant correlations are left blank. Significance levels are indicated by asterisks (* *p* < 0.05, ** *p* < 0.01, *** *p* < 0.001). The color scale ranges from dark red (strong positive correlation, r = +1) to dark blue (strong negative correlation, r = –1). The strongest positive correlation was observed between all comorbidities and hypertension (r = 0.74, *p* < 0.001), since the one is part of the other, and in hospitalization days and hospitalization status (r = 0.57, *p* < 0.001), which does not need explanation. Brixia score showed statistically significant positive associations with hospitalizations (r = 0.37, *p* < 0.001), number of days (r = 0.23, *p* < 0.001) and gender (r = 0.17, *p* < 0.05). Age expectedly demonstrated consistent positive correlations with multiple variables, including all comorbidities (r = 0.41, *p* < 0.001), hypertension (r = 0.35, *p* < 0.001), diabetes (r = 0.2, *p* < 0.01), hospitalization days (r = 0.35, *p* < 0.001) and hospital admission (r = 0.3, *p* < 0.001). Older patients were more prone to negative health developments. Gender showed only weak positive associations, most notably with hospitalization status (r = 0.18, *p* < 0.01), suggesting a mild male predominance among patients that were hospitalized. Hypertension, as the single most frequent comorbidity in the patient group, was correlated with hospitalization days (r = 0.21, *p* < 0.01) and hospitalization (r = 0.30, *p* < 0.001). Hypertension and diabetes occurred together in more than 10% of patients and the correlation coefficient between them was also notable (r = 0.34, *p* < 0.001) (Figure 4).

### 3.4. Machine Learning Classification Models

**Figure 5:**
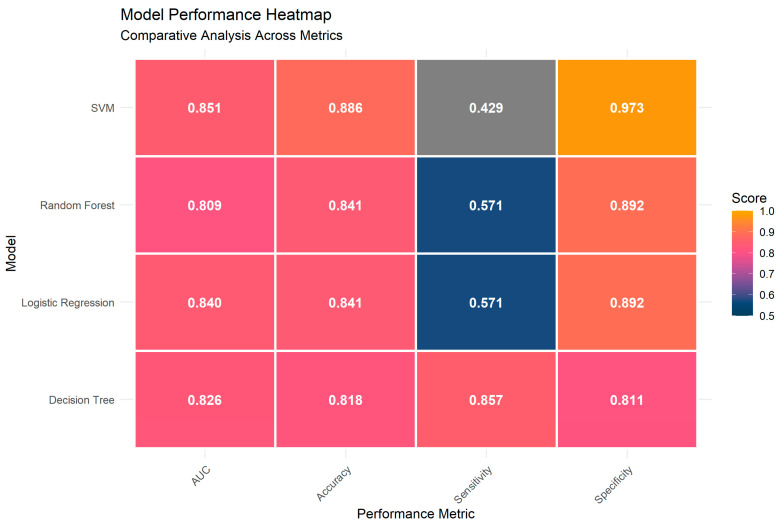
Comparative performance heatmap of four machine learning models (Decision Tree, Logistic Regression, Random Forest, and Support Vector Machine—SVM) for predicting hospitalization in COVID-19 patients (*n* = 222), using formula: HOSPITALIZED~BRIXIA.SCORE + GENDER + AGE + HYPERTENSION + DIABETES. Color scale represents performance scores (0.5 = blue to 1.0 = orange). Legend: AUC = Area Under the Receiver Operating Characteristic Curve.

While DT and LR are relatively easy to interpret, RF and SVM belong to the group of so-called “black box” algorithms, whose internal logic is not driven by formulas that humans can follow to understand the modelling results. All four models provided “good” prediction capabilities (AUC > 0.8), satisfactory accuracy (i.e., the proportion of all patients correctly classified as “hospitalized” or “non-hospitalized”), high specificity (the proportion of patients correctly predicted as “non-hospitalized” among those who did not require hospitalization), and variable sensitivity results (the proportion correctly predicted as “hospitalized” among those who required hospitalization) (Figure 5, Appendix A). The classification power of SVM for correctly predicting patients who did not need hospitalization was the highest (specificity = 0.973), contributing to high model accuracy and high AUC values, while compromising correct predictions for hospitalization necessity. The most robust model was the DT, which appeared to be reliable in correctly predicting the outcome of disease development. DT showed the best balance between the identification of cases that needed to be hospitalized (sensitivity = 0.857) and those who needed to be sent home for treatment continuation (specificity = 0.811).

### 3.5. Determination of the Most Important Variables in Classification Modeling

**Figure 6:**
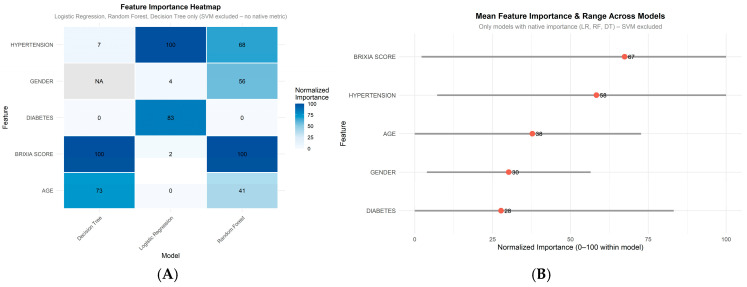
Feature importance comparison across machine learning models. (**A**) Heatmap showing normalized feature importance scores (0–100 scale within each model) for the five main predictors. Darker blue indicates higher relative importance within that model. (**B**) Mean normalized feature importance is marked by a red dot and the range of importance values are depicted with a grey horizontal line across the models that provides native feature importance scores (Decision Tree, Random Forest, and Logistic Regression; SVM excluded). Features are ranked by mean importance. Higher values indicate higher contribution to model decisions.

Different machine learning models provide various measures of how important each predictor variable can be for the outcome. LR has the *magnitude of the coefficients* that indicates the strength of association between a predictor and the likelihood of hospitalization. RF has variable importance that is assessed using *MeanDecreaseAccuracy*, which reflects how much the overall model accuracy lowers when a given variable is removed. DT determines variable importance through how often and how effectively each variable is used for splitting the data, while SVM cannot directly measure variable importance, so it was omitted. Features (Brixia score, gender, age, diabetes and hypertension) importance comparison across predictive models has been made joining the results from three models (Figure 6). Brixia score appears to be the most important variable in the process of predicting the disease outcome (course of the disease) in the terms of hospitalization or home treatment. It was followed by hypertension, diabetes, age and gender.

Heatmap of feature importance scores (0–100 scale) across three ML models in predicting hospitalization (A) and consistency of feature importance across ML models: mean importance score (0–100 scale) with min–max range for prediction of hospitalization in COVID-19 patients (B).

## 4. Discussion

### 4.1. Radiological Modalities, Brixia Score and Importance of Comorbidities for the Outcome

Numerous radiological diagnostics methods have been applied to more accurately diagnose lung damage during the corona disease. Computed tomography (CT) of the lung and computed tomography angiography (CTA) were represented as choice modality in patients with suspected complications such as pulmonary thromboembolism, but standard radiography of the chest requires less time for examination and lower dose exposure allowed rapid orientation on the existence of interstitial or alveolar consolidation in the lung parenchyma using Brixia score [9,10]. Several studies focused on the evaluation of chest X-rays during COVID-19 using the Brixia score, giving importance to this scoring system that could also be used as a predictive model. The study by *Sofic* et al. (*2022*) [11] showed that the Brixia score has a statistically significant correlation with the values of D dimer and C reactive protein (CRP), while the study of *Hanley* et al. (*2022*) [12] concluded that a higher value of the Brixia score was associated with intubation, the need for mechanical ventilation, and an increased risk of death. It is shown that patients with a high Brixia score and additional factor such as older age or immunosuppression will be likely related to in-hospital death [13]. In the study *Boari* et al. (*2020*) [14] expressed the fact that the presence of hypertension, diabetes mellitus or underlying cardiac disease was associated with a worse outcome of the patients.

More than 98% of patients with some comorbidities were hospitalized, which is 29% more than in groups without comorbidities. If comorbidities such as hypertension, diabetes, obesity, etc., will be considered as unique predictors, as well as other data as age, gender, etc., Brixia score will outperform any single one (Figure 6). In the framework of robust outcome prediction with machine learning algorithm, hypertension as the predictor is very close to Brixia score in the context of hospitalization of COVID-19 patients, followed by diabetes, age and gender. Overall, best prediction of outcome will be made using all possible patient data.

### 4.2. Applied ML Algorithms

If all these four models are compared, it is observed that the highest sensitivity was demonstrated by the DT model (0.857), in relation to SVM (0.429), RF (0.57) and Logistic Regression (0.571). This high sensitivity is of great importance in the context of COVID-19, where the primary clinical goal may be to minimize false negatives (e.g., avoid missing patients who will require hospitalization). Early identification of patients that are at high risk for hospitalization based on readily available characteristics such as the Brixia score favors the use of DT, which may be a useful tool for triage of patients during a respiratory virus pandemic. While the DT achieved the best sensitivity, it showed the lowest specificity (0.811) compared to the other applied models. In contrast, SVM exhibited extremely high specificity (0.973), but low sensitivity (0.429). Logistic Regression and Random Forest showed average performance (specificity 0.892, sensitivity 0.571), representing a more balanced but still cautious profile (Figure 5, Appendix B). Those differences show that the best model depends on clinical priorities. Decision Tree (DT) should be selected when the main goal may be to avoid missing any patients who truly need hospitalization (high sensitivity). On the other hand, Support Vector Machine (SVM) should be selected when the priority is to prevent unnecessary hospital admissions (high specificity).

DT is also the most clinically transparent model that directly exposes the hierarchical decision rules (e.g., Brixia score threshold → hypertension → age). The transparency provided by DT gives clinicians the advantage of understanding the reasoning that results from the use of such predictive models. Although SVM revealed the highest AUC (0.851) among the linear/non-linear models tested, its “black box” nature may reduce application among clinicians due to limitations in the explainability of the method.

A limitation of the feature importance visualization is the use of model-specific importance metrics. Rescaling to a 0–100 range within models allows visual consideration of ranking consistency but does not preserve the original metric units or enable direct quantitative comparison. Heatmap and mean importance plot are more qualitative indicators of feature relevance across methods rather than precise measures. Having that in mind, feature importance analysis indicated that the Brixia score consistently ranked as the most influential predictor across nearly all models (mean importance 61/100, highest in RF and DT, Figure 6).

Both tree-based approaches (DT and RF) processed a mixed set data (binary comorbidities, Brixia score, continuous age and hospitalization days) more efficiently than the linear SVM and Logistic Regression models.

The DT, with its high sensitivity, interpretability, clarity and computational simplicity, appears best suited for deployment in conditions that require high level of explainability.

### 4.3. Study Perspectives

COVID-19 crisis is accelerator that just raised natural and essential importance of medicine among wider public. Machine learning as the by-product of universal digitalization of world should be harnessed in favor of humanity. Its application in medicine is slower than in, e.g., e-commerce, but has potential that justifies the necessary efforts.

Using all available patients and all patient data would be the best usage scenario for machine learning algorithms, such as the four methods used in this study. In that sense, further integration and aggregation of databases will allow wider deployment of SVM, RF, LR and DT and similar algorithms in various clinical branches in medicine. Regarding current study, larger number of non-hospitalized patients would allow better predicting performance of the machine learning algorithms.

Regarding study limitations of this study, imbalance in the number of male versus female patient cohorts (155 versus 67), may put some constraints to the universality of results and some future study will benefit from more balanced gender cohorts.

Furthermore, employing models to predict patient outcome and in that sense fine-tune medical care, machine learning models have potential in other medical branches including radiology. Random forest classifiers proved to be the best choice in separating radiology reports with some specific “actionable” findings from the others [15]. ML methods were successfully tested with certain degree of preciseness (when the selection of radiology subspecialist for specific patients was referent point) in automatic assignment of protocols for CT and MRI [16]. Break-throughs in machine learning reflected through deep learning and its class convolutional neural network may be followed by wider application of such methods in radiology through classification (detecting lesions in medical images), segmentation (depicting organ volume and shape) and detection (detection of abnormalities among large number of images, may be useful for large screening projects) [17]. Application of ML in the context of clinical data (such as COVID-19) can be useful during treatment, but also in cases such as understanding of future complications and probability of their occurrence.

## 5. Conclusions

- Comorbidities, Brixia score, age and gender were significantly correlated with hospitalization among COVID-19 patients.
- Hypertension was the most influential single comorbidity factor.
- Brixia scoring has proven to be a significant radiological tool for analysis of chest X-ray images.
- Three ML models provided “good” prediction capabilities (AUC > 0.8), while SVM despite high AUC had very low sensitivity, and emphasize Brixia score as the strongest predictor of hospitalization, followed by hypertension, age, gender and diabetes.
- DT as the most clinically transparent model provided the best balance between sensitivity and specificity.

## Figures and Tables

**Figure A1:**
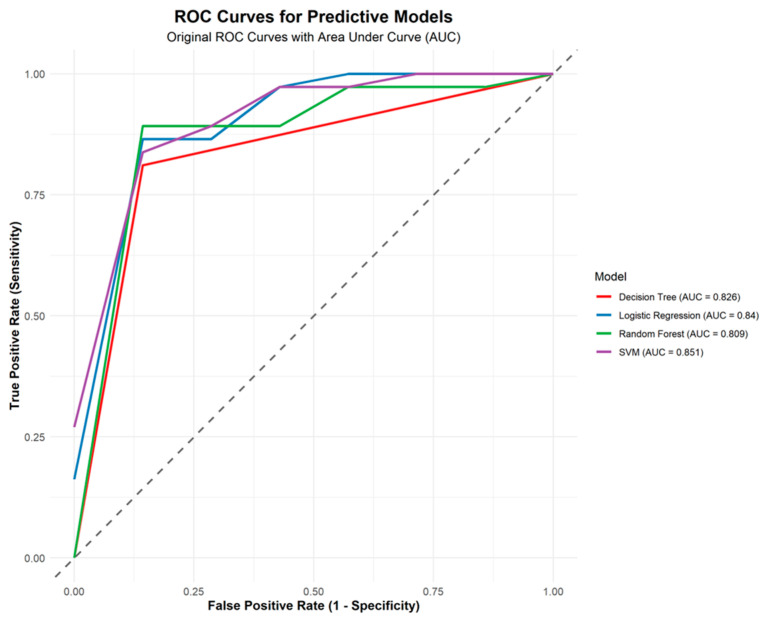
ROC curve for classification model (AUROC = 0.823) (0.6–0.7 = “poor”, 0.7–0.8 = “fair”, 0.8–0.9 = “good”, 0.9–1.0 = “excellent”).

**Figure A2:**
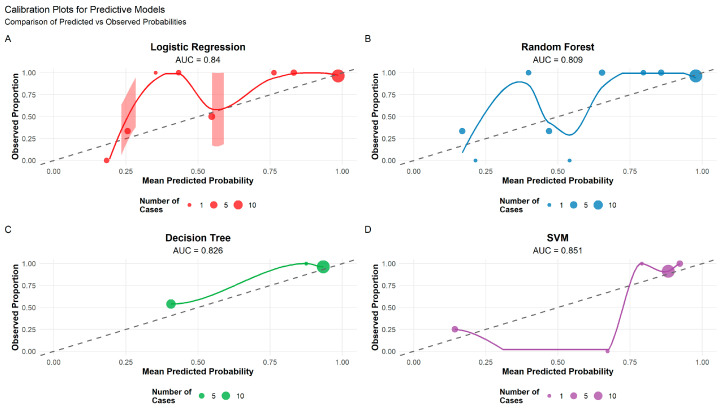
Calibration plots comparing predicted versus observed probabilities of hospitalization in COVID-19 patients (*n* = 222) for four machine learning models: (**A**) Logistic Regression (AUC = 0.840), (**B**) Random Forest (AUC = 0.809), (**C**) Decision Tree (AUC = 0.826), and (**D**) Support Vector Machine (SVM; AUC = 0.851). In each panel, the x-axis represents the mean predicted probability of hospitalization and the y-axis the observed proportion of hospitalized patients. The dashed diagonal line indicates perfect calibration.

## Data Availability

The original contributions presented in this study are included in the article/Supplementary Materials. Further inquiries can be directed to the corresponding author(s).

## Supplementary Materials

The following supporting information can be downloaded at https://www.mdpi.com/article/10.3390/medicina62020392/s1; Table S1: Patient Characteristics of the Study Cohort.

## Abbreviations

The following abbreviations are used in this manuscript:

UCCV	University Clinical Centre of Vojvodina
PACS	Picture Archiving and Communication System
RIS	Radiology Information System
CIS	Clinical Information System
ML	Machine learning
DT	Decision Tree
LR	Logistic Regression
RF	Random Forest
SVM	Support Vector Machine
ROC	Receiver Operating Characteristic
