# Supplementary material for: Using Machine Learning Methods to Predict Hospitalization Based on Brixia Score and Patient Clinical Data (from the COVID-19 Pandemic)
Source: Medicina (Kaunas). 2026 Feb 17;62(2):392. doi: 10.3390/medicina62020392 (PMC12942398; doi:10.3390/medicina62020392)
Supplement: Supplementary file 1 [file medicina-62-00392-s001.zip › medicina-4123485-supplementary.pdf]

Supplement Table S1. Patient Characteristics of the Study Cohort

GENDER	AGE	ALL COMORBIDITIES	HYPERTENSION	DIABETES	BRIXIA SCORE	HOSPITALIZATION DAYS	HOSPITALIZED
MALE	29	YES	NO	YES	12	9	YES
MALE	67	NO	NO	NO	16	14	YES
MALE	69	NO	NO	NO	10	6	YES
MALE	40	YES	NO	NO	8	5	YES
MALE	61	YES	YES	YES	14	3	YES
FEMALE	68	YES	YES	NO	10	21	YES
MALE	42	YES	NO	NO	6	7	YES
FEMALE	70	NO	NO	NO	2	6	YES
FEMALE	80	NO	NO	NO	4	4	YES
MALE	50	NO	NO	NO	6	3	YES
FEMALE	73	YES	NO	YES	16	21	YES
FEMALE	69	YES	NO	NO	3	7	YES
FEMALE	41	NO	NO	NO	2	0	NO
MALE	72	YES	YES	NO	9	4	YES
FEMALE	64	NO	NO	NO	13	4	YES
FEMALE	35	NO	NO	NO	1	0	NO
MALE	28	NO	NO	NO	16	7	YES
MALE	32	NO	NO	NO	3	0	NO
FEMALE	30	NO	NO	NO	1	0	NO
MALE	54	YES	YES	NO	8	9	YES
FEMALE	42	NO	NO	NO	1	0	NO
FEMALE	36	NO	NO	NO	0	0	NO
MALE	42	NO	NO	NO	14	14	YES
MALE	38	NO	NO	NO	10	6	YES
MALE	51	NO	NO	NO	4	5	YES
MALE	37	NO	NO	NO	9	5	YES
MALE	50	NO	NO	NO	3	6	YES

MALE	48	NO	NO	NO	9	11	YES
MALE	73	YES	NO	NO	16	11	YES
FEMALE	62	YES	NO	YES	10	11	YES
MALE	61	YES	YES	YES	4	5	YES
MALE	60	YES	YES	YES	5	11	YES
MALE	55	NO	NO	NO	1	0	NO
MALE	84	YES	YES	NO	5	3	YES
MALE	64	YES	YES	NO	7	7	YES
MALE	55	YES	NO	NO	0	0	NO
MALE	29	NO	NO	NO	2	4	YES
MALE	79	YES	NO	NO	10	7	YES
FEMALE	82	YES	YES	NO	0	11	YES
MALE	86	YES	NO	NO	1	12	YES
MALE	51	NO	NO	NO	0	11	YES
FEMALE	72	YES	YES	YES	3	3	YES
MALE	47	NO	NO	NO	4	0	NO
MALE	65	NO	NO	NO	14	18	YES
FEMALE	78	NO	NO	NO	5	0	NO
FEMALE	52	NO	NO	NO	0	0	NO
FEMALE	76	YES	YES	NO	10	15	YES
FEMALE	49	YES	YES	NO	0	9	YES
MALE	38	NO	NO	NO	6	0	NO
MALE	23	NO	NO	NO	0	0	NO
MALE	47	YES	YES	NO	10	8	YES
MALE	64	YES	YES	YES	1	4	YES
MALE	74	YES	YES	NO	2	13	YES
MALE	62	YES	YES	NO	12	7	YES
FEMALE	58	NO	NO	NO	4	8	YES
FEMALE	74	YES	NO	NO	0	8	YES
FEMALE	60	NO	NO	NO	2	10	YES

FEMALE	57	YES	YES	YES	8	11	YES
FEMALE	68	NO	NO	NO	0	0	NO
MALE	57	NO	NO	NO	5	12	YES
FEMALE	37	NO	NO	NO	1	0	NO
MALE	50	NO	NO	NO	6	8	YES
MALE	67	YES	YES	NO	16	8	YES
MALE	56	NO	NO	NO	3	0	NO
MALE	56	NO	NO	NO	6	25	YES
MALE	78	YES	NO	NO	8	19	YES
MALE	56	NO	NO	NO	12	11	YES
MALE	42	NO	NO	NO	0	0	NO
MALE	40	NO	NO	NO	2	0	NO
FEMALE	56	NO	NO	NO	1	0	NO
MALE	43	NO	NO	NO	2	0	NO
FEMALE	38	NO	NO	NO	6	0	NO
MALE	80	YES	YES	NO	2	18	YES
MALE	40	NO	NO	NO	2	0	NO
MALE	58	YES	NO	NO	2	11	YES
MALE	73	YES	NO	NO	9	17	YES
FEMALE	80	NO	NO	NO	14	16	YES
MALE	73	YES	YES	NO	6	0	NO
MALE	57	YES	YES	NO	8	11	YES
FEMALE	25	NO	NO	NO	2	0	NO
MALE	43	NO	NO	NO	2	0	NO
MALE	37	NO	NO	NO	7	5	YES
MALE	29	NO	NO	NO	5	3	YES
MALE	42	NO	NO	NO	1	12	YES
MALE	32	NO	NO	NO	2	20	YES
MALE	65	NO	NO	NO	6	11	YES
MALE	78	YES	YES	YES	8	40	YES

MALE	63	YES	YES	NO	9	7	YES
FEMALE	55	YES	YES	YES	8	10	YES
FEMALE	25	NO	NO	NO	1	0	NO
MALE	49	NO	NO	NO	0	0	NO
MALE	64	NO	NO	NO	1	0	NO
MALE	45	NO	NO	NO	1	0	NO
MALE	64	NO	NO	NO	5	12	YES
MALE	60	YES	YES	NO	4	5	YES
MALE	60	NO	NO	NO	5	20	YES
MALE	65	YES	YES	YES	6	12	YES
MALE	48	YES	NO	NO	6	16	YES
MALE	80	YES	YES	NO	5	15	YES
FEMALE	66	NO	NO	NO	8	16	YES
MALE	36	NO	NO	NO	3	0	NO
MALE	42	NO	NO	NO	4	11	YES
FEMALE	48	YES	YES	NO	6	14	YES
MALE	69	YES	YES	YES	1	15	YES
FEMALE	86	NO	NO	NO	6	15	YES
MALE	63	NO	NO	NO	5	10	YES
MALE	66	YES	NO	NO	0	8	YES
MALE	45	YES	NO	NO	1	18	YES
FEMALE	24	NO	NO	NO	1	0	NO
MALE	32	NO	NO	NO	3	8	YES
FEMALE	39	NO	NO	NO	1	7	YES
MALE	55	YES	YES	NO	4	10	YES
MALE	58	NO	NO	NO	5	12	YES
MALE	21	NO	NO	NO	3	8	YES
FEMALE	54	YES	YES	NO	1	9	YES
MALE	59	YES	YES	NO	3	14	YES
MALE	49	NO	NO	NO	5	11	YES

FEMALE	48	YES	NO	NO	3	21	YES
MALE	35	YES	YES	NO	1	5	YES
FEMALE	67	YES	YES	YES	8	11	YES
MALE	84	YES	NO	NO	4	47	YES
FEMALE	84	NO	NO	NO	3	6	YES
MALE	50	YES	NO	NO	1	12	YES
FEMALE	54	NO	NO	NO	2	0	NO
MALE	68	NO	NO	NO	4	0	NO
MALE	72	YES	NO	YES	3	11	YES
FEMALE	50	NO	NO	NO	6	12	YES
FEMALE	35	NO	NO	NO	6	8	YES
FEMALE	61	NO	NO	NO	1	0	NO
MALE	57	NO	NO	NO	1	0	NO
FEMALE	39	NO	NO	NO	1	0	NO
MALE	65	NO	NO	NO	0	9	YES
MALE	67	YES	NO	NO	3	9	YES
MALE	70	YES	YES	YES	2	13	YES
MALE	68	NO	NO	NO	7	17	YES
FEMALE	65	YES	NO	NO	1	6	YES
MALE	46	YES	YES	NO	1	12	YES
FEMALE	72	NO	NO	NO	2	13	YES
MALE	43	NO	NO	NO	2	12	YES
MALE	43	NO	NO	NO	4	13	YES
FEMALE	53	YES	YES	NO	4	21	YES
FEMALE	68	NO	NO	NO	4	12	YES
MALE	41	NO	NO	NO	7	11	YES
FEMALE	71	NO	NO	NO	3	11	YES
FEMALE	70	YES	YES	NO	3	17	YES
MALE	56	YES	YES	NO	7	12	YES
MALE	65	YES	YES	YES	5	11	YES

FEMALE	24	NO	NO	NO	2	0	NO
MALE	54	NO	NO	NO	7	10	YES
MALE	39	NO	NO	NO	2	7	YES
MALE	83	YES	YES	YES	4	13	YES
MALE	59	YES	YES	YES	1	7	YES
MALE	59	NO	NO	NO	7	17	YES
MALE	43	YES	NO	NO	1	34	YES
FEMALE	73	YES	YES	YES	6	8	YES
MALE	47	NO	NO	NO	8	20	YES
MALE	60	NO	NO	NO	5	20	YES
MALE	25	NO	NO	NO	8	10	YES
MALE	52	NO	NO	NO	6	9	YES
MALE	68	NO	NO	NO	0	10	YES
MALE	52	NO	NO	NO	6	16	YES
MALE	41	YES	NO	NO	0	8	YES
FEMALE	53	YES	YES	NO	6	19	YES
MALE	64	YES	YES	YES	2	29	YES
FEMALE	70	YES	YES	NO	1	12	YES
FEMALE	65	NO	NO	NO	0	14	YES
MALE	82	YES	YES	NO	4	59	YES
MALE	82	YES	YES	NO	6	19	YES
FEMALE	47	NO	NO	NO	8	24	YES
MALE	67	YES	YES	NO	5	17	YES
MALE	63	YES	NO	YES	9	17	YES
MALE	67	YES	YES	YES	5	13	YES
FEMALE	64	NO	NO	NO	9	12	YES
MALE	75	YES	YES	NO	4	24	YES
MALE	81	YES	YES	NO	1	13	YES
FEMALE	59	YES	YES	YES	1	18	YES
MALE	63	YES	NO	NO	0	8	YES

MALE	67	YES	YES	NO	1	23	YES
FEMALE	26	YES	NO	NO	1	11	YES
FEMALE	31	NO	NO	NO	6	13	YES
MALE	71	YES	YES	NO	9	14	YES
MALE	72	YES	YES	NO	2	13	YES
MALE	70	YES	YES	NO	3	9	YES
MALE	32	NO	NO	NO	1	5	YES
FEMALE	44	YES	YES	NO	1	9	YES
MALE	36	NO	NO	NO	6	13	YES
MALE	36	NO	NO	NO	2	20	YES
MALE	50	NO	NO	NO	4	9	YES
MALE	46	NO	NO	NO	7	15	YES
MALE	38	NO	NO	NO	13	18	YES
FEMALE	71	YES	YES	YES	6	7	YES
FEMALE	65	YES	YES	NO	6	9	YES
MALE	61	YES	YES	NO	9	10	YES
MALE	70	YES	YES	NO	5	24	YES
MALE	79	YES	YES	NO	9	10	YES
MALE	70	YES	YES	NO	7	10	YES
MALE	51	YES	YES	YES	11	13	YES
MALE	45	YES	YES	NO	14	10	YES
MALE	68	YES	YES	NO	13	16	YES
FEMALE	52	NO	NO	NO	3	7	YES
FEMALE	64	NO	NO	NO	11	14	YES
MALE	56	YES	NO	YES	7	12	YES
MALE	54	YES	YES	NO	8	10	YES
FEMALE	51	YES	YES	NO	6	13	YES
MALE	40	NO	NO	NO	8	14	YES
MALE	33	NO	NO	NO	13	10	YES
MALE	50	YES	NO	NO	5	7	YES

MALE	51	NO	NO	NO	0	8	YES
MALE	36	YES	YES	YES	4	16	YES
MALE	78	YES	NO	YES	10	11	YES
MALE	54	YES	YES	NO	4	11	YES
FEMALE	66	YES	YES	NO	6	20	YES
MALE	44	YES	YES	NO	6	8	YES
MALE	70	NO	NO	NO	14	42	YES
MALE	39	NO	NO	NO	5	6	YES
MALE	58	YES	NO	YES	8	11	YES
MALE	65	YES	YES	YES	10	13	YES
MALE	36	YES	YES	NO	6	8	YES
MALE	63	NO	NO	NO	8	16	YES
MALE	40	YES	YES	NO	13	9	YES
MALE	24	NO	NO	NO	15	8	YES
MALE	68	NO	NO	NO	7	14	YES
